# Chenodeoxycholic Acid Reduces Hypoxia Inducible Factor-1α Protein and Its Target Genes

**DOI:** 10.1371/journal.pone.0130911

**Published:** 2015-06-22

**Authors:** Yunwon Moon, Su Mi Choi, Soojeong Chang, Bongju Park, Seongyeol Lee, Mi-Ock Lee, Hueng-Sik Choi, Hyunsung Park

**Affiliations:** 1 Department of Life Science, University of Seoul, Seoul, Korea; 2 College of Pharmacy, Seoul National University, Seoul, Korea; 3 National Creative Research Initiatives Center for Nuclear Receptor Signals and Hormone Research Center, School of Biological Sciences and Technology, Chonnam National University, Gwangju, Korea; IRCCS Istituto Oncologico Giovanni Paolo II, ITALY

## Abstract

This study evaluated HIF-1α inhibitors under different hypoxic conditions, physiological hypoxia (5% O_2_) and severe hypoxia (0.1% O_2_). We found that chenodeoxy cholic acid (CDCA) reduced the amount of HIF-1α protein only under physiological hypoxia but not under severe hypoxia without decreasing its mRNA level. By using a proteasome inhibitor MG132 and a translation inhibitor cyclohexamide, we showed that CDCA reduced HIF-1α protein by decreasing its translation but not by enhancing its degradation. The following findings indicated that farnesoid X receptor (FXR), a CDCA receptor and its target gene, Small heterodimer partner (SHP) are not involved in this effect of CDCA. Distinctly from CDCA, MG132 prevented SHP and an exogenous FXR agonist, GW4064 from reducing HIF-1α protein. Furthermore a FXR antagonist, guggulsterone failed to prevent CDCA from decreasing HIF-1α protein. Furthermore, guggulsterone by itself reduced HIF-1α protein even in the presence of MG132. These findings suggested that CDCA and guggulsterone reduced the translation of HIF-1α in a mechanism which FXR and SHP are not involved. This study reveals novel therapeutic functions of traditional nontoxic drugs, CDCA and guggulsterone, as inhibitors of HIF-1α protein.

## Introduction

Hypoxia up-regulates the transcription of genes involved in anaerobic ATP production, oxygen supply, inflammation, metastasis, and angiogenesis. Hypoxia-inducible factor-1 (HIF-1) is a master transcription factor which stimulates hundreds of hypoxia-inducible genes including vascular endothelial growth factor (VEGF), erythropoietin, inflammatory cytokines, glucose transporters and glycolytic enzymes. HIF consists of HIF-α and β subunits which belong to the basic helix-loop-helix Per-Arnt-Sim family.

The amount of HIF-1α protein is determined by balance between its degradation and translation [1, 2]. Oxygen concentration influences both degradation and translation of HIF-1α. Hydroxylation of proline-564 and/or 402 residues of human HIF-1α initiates its ubiquitination and subsequent proteasomal degradation [3]. Prolyl hydroxylation of HIF-1α is catalyzed by a novel HIF-α-specific prolyl hydroxylase, which requires O_2_, α-ketoglutarate, vitamin C, and Fe^2+^ [4–6]. Von Hippel Lindau tumor suppressor, which functions as an E3 ubiquitin ligase, interacts with the hydroxylated proline residues of HIF-1α and brings about assembly of a complex that activates an ubiquitin-dependent proteasome [7–9]. Therefore under normoxic condition, HIF-1α protein is degraded by hydroxylation-dependent ubiquitin system, whereas under hypoxia, proline hydroxylation ceases and HIF-1α protein accumulates. In mammalian cells, a family of HIF-α-specific prolyl-4 hydroxylases have been identified and given the acronyms PHD1 (EGLN2), PHD2 (EGLN1), and PHD3 (EGLN3) [10–12]. Among three, PHD2 is the primary enzyme to hydroxylate HIF-1α protein [10, 13–15]. To be a functional transactivator, the stabilized HIF-1α should be able to recruit its coactivator, CBP/p300. Asparagine 803 of HIF-1α is also hydroxylated by an asparagine hydroxylase, referred to as Factor-Inhibiting HIF-1 (FIH-1). The hydroxylated asparagine residue prevents HIF-1α from binding CBP/p300. A lack of oxygen reduces the activities of both PHDs and FIH-1, so stabilizing the transactive form of HIF-1α [16, 17]. The findings that Km values of PHDs and FIH-1 for O_2_ are close to oxygen concentrations of normal peripheral tissues in a body which is estimated from 2% to 8% suggest that hydroxylation of HIF-1α protein can be sensitively changed in the normal peripheral tissues [3, 10, 13, 18–20]. The facts that these hydroxylases can be inhibited by depletion of reducing agents, vitamin C and Fe^2+^ and by increase of their product succinate, suggest that strong oxidants such as reactive oxygen species and succinate can increase HIF-1α activities [21].

In addition to hydroxylation-dependent ubiquitination, the translation of HIF-1α protein is also regulated by hypoxia. Only severe hypoxia but not physiological hypoxia (> 3% oxygen) causes unfolded protein response (UPR) which inhibits Cap-dependent translation [22]. However severe hypoxia does not reduce *de novo* synthesis of HIF-1α protein by activating Cap-independent translation of HIF-1α. Reflecting the findings that both severe and physiological hypoxia prevent hydroxylation-dependent degradation but that only severe hypoxia increases Cap-independent translation of HIF-1α, it is necessary to distinguish severe hypoxia and physiological hypoxia. Distinctly from many previous studies, this study investigated HIF-1α inhibitors under two hypoxic conditions, 5% oxygen and 0.1% oxygen.

Recently, Liver-specific HIF-1α knock-out revealed the importance of HIF-1α in metabolic diseases. Partial pressure of O_2_ in a normal liver was estimated at 2% to 8% suggesting that HIF-1α protein is partially activated in the normal liver. In addition, HIF-1α protein can be further activated in several liver diseases, either by increasing oxygen consumption or by producing HIF-1α activators such as reactive oxygen species [23]. HIF-1α enhances the hepatic inflammation, fibrosis and cancer by inducing its target genes such as vascular endothelial growth factor, plasminogen activator inhibitor-1, prolyl 4-hydroxylase, alpha peptide 1 (P4HA1), lysyl oxidase (LOX) and endoplasmic reticulum oxidoreductin 1-like (ERO1L) [24–26]. Liver specific deletion of HIF-1α alleviated alcohol-induced liver damage by reducing lipid accumulation and inflammation [27]. CDCA, a major component of bile acids has been used as a safe medicine to improve liver diseases. This study investigated whether CDCA reduces HIF-1α protein in liver cells under 5% as well as 0.1% oxygen.

Bile acids function not only as detergents for absorption of lipids but also as hormones that activates three nuclear receptors, farnesoid X receptor (FXR, NR1H4), pregnane X receptor, and vitamin D receptor and a G protein coupled receptor, TGR5. Among them, FXR is a specific receptor for CDCA.

Bile acid synthesis is tightly regulated by a negative feedback mechanism. As bile acid levels increase, FXR is activated and induces the transcription of short heterodimer protein (SHP; NR0B2). Then SHP inhibits the transactivation ability of LRH-1 which enhances the expression of both cytochromeP450 7a1(Cyp7a1) and SHP. Thereby SHP represses the expression of both Cyp7a1 and itself. Thus, SHP plays a key role in the negative feedback regulation of bile acid synthesis. SHP is an orphan receptor that contains ligand-binding and transcription regulation domain but lacks a DNA binding domain. SHP interacts with thyroid receptor, retinoic acid receptors and estrogen receptor by inhibiting their DNA binding ability [28, 29]. SHP also modulates transactivation of interacting target proteins by occupying coactivator binding sites or by recruiting corepressors [30–33]. The findings that HIF-1α enhances the hepatic inflammation, fibrosis and cancer, and that CDCA and FXR agonists improve liver function lead us to investigate whether CDCA and a FXR agonist inhibit HIF-1α function in liver cells.

## Materials and Methods

### Materials

Chenodeoxycholic acid (CDCA), GW4064 and anti-β-actin antibody were purchased from Sigma-Aldrich (St. Louis, MO, USA). Guggulsterone (Z form) was purchased from Enzo Life Sciences (Farmingdale, NY, USA). Anti-HIF-1α was purchased from BD Biosciences (Palo Alto, CA, USA). The anti-SHP, anti-HDAC1 and anti-14-3-3γ antibodies were purchased from Santa Cruz Biotechnology (Dallas, TX, USA). We used the human cDNAs of SHP (human, NM_021969) for the transfection assays [34].

### Cells and treatments

**Fig 1:**
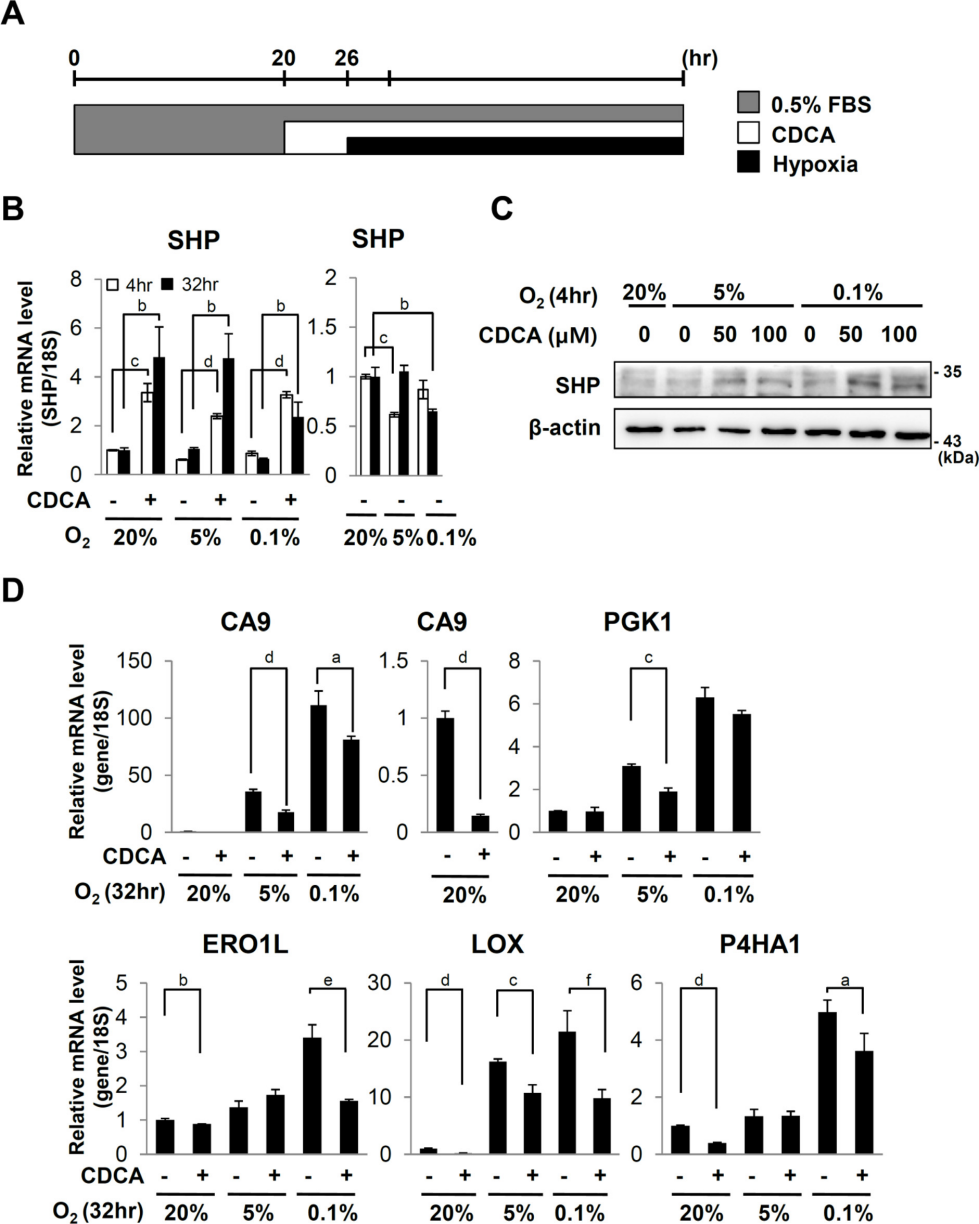
Effect of CDCA on hypoxic target genes. (A) Experimental scheme. HepG2 cells were serum starved with medium containing 0.5% FBS for 20 hours prior to CDCA (100 μM or indicated dose) treatment. 6 hours after CDCA treatment, the cells were exposed to 20%, 5% or 0.1% O_2_ for the indicated hours. (B) Quantitative RT-PCR analyses of SHP mRNA. The expression level was normalized with the expression level of 18s rRNA. (C) Western analyses for SHP and β-actin. β-actin protein was detected as a loading control. Data shown are representative of three experiments (C) Quantitative RT-PCR analyses of carbonic anhydrase 9 (CA9), phosphoglycerate kinase1 (PGK1), endoplasmic reticulum oxidoreductin 1-like (EROL1), lysyl oxidase (LOX), prolyl 4-hydroxylase, alpha peptide 1 (P4HA1). a, *p* ≤ 0.1; b, *p* ≤ 0.05; c, *p* ≤ 0.01; d, *p* ≤ 0.001; e, *p* = 0.197; f, *p* = 0.724.

Human HepG2 cells were purchased from American Type Culture Collection. HepG2 cells were cultured in MEM containing non-essential amino acids and 10% fetal bovine serum (FBS) (Lonza, Basel, Switzerland) in humidified air containing 5% CO_2_ at 37°C. Prior to CDCA, GW4064 or Guggulsterone treatments, HepG2 cells were serum starved with medium containing 0.5% FBS for 20 hours. We pretreated cells with CDCA, GW4064 or Guggulsterone for 6 hours before hypoxic exposure (Fig 1A). The cells were exposed to hypoxic conditions (0.1% or 5% O_2_) by incubation in an anaerobic incubator (Model 1029, Forma Scientific, Inc., Marietta, OH, USA) or in an In Vivo200 Hypoxia Workstation (Ruskinn Technology, Leeds, UK).

### Quantitative real-time reverse transcription (RT)-polymerase chain reaction (PCR) (qRT-PCR)

One microgram of isolated total RNA was used for reverse transcription. The quantitative RT-PCR was performed using the 7000 sequence detection system (Applied Biosystems, Warrington, UK) with Power SYBR Green Master Mix reagent (Applied Biosystems) as described [35]. The expression level of 18S rRNA was used for normalization. The following forward and reverse primers were used for the specific PCR primers: human SHP; 5’- AGATGTTGACATCGCTGGCCTTCT-3’ and 5’-AGAGCTGTTCCTAAGGAGCCAAGT-3’ (GenBank Accession Number NM_021969); human HIF-1α; 5’-TTGGCAGCAACGACACAGAAACTG-3’ and 5’- TTGAGTGCAGGGTCAGCACTACTT-3’ (GenBank Accession Number NM_001530); human CA9; 5′-CAGTTGCTGTCTCGCTTGGA-3′ and 5′-TGAAGTCAGAGGGCAGGAGTG-3′ (GenBank Accession Number NM_001216.2); human PGK1; 5’-TTAAAGGGAAGCGGGTCGTTA-3’ and 5’-TCCATTGTCCAAGCAGAATTTGA-3’ (GenBank Accession Number NM_000291.3); human ERO1L; 5’- GCCCGTTTTATGCTTGATGT-3’ and 5’-AACTGGGTATGGTGGCAGAC-3’ (GenBank Accession Number NM_014584.1); human LOX; 5’-ACAGGGATTGAGTCCTGGCTGTTA-3’ and 5’-TGAGGCATACGCATGATGTCCTGT-3’ (GenBank Accession Number AF039291); human P4HA1; 5’- GGCAGCCAAAGCTCTGTTAC-3’ and 5’- AAAGCAGTCCTCAGCCGTTA-3’ (GenBank Accession Number NM_000917.3); human 18S rRNA; 5′-ACCGCAGCTAGGAATAATGGAATA-3′ and 5′-CTTTCGCTCTGGTCCGTCTT-3′ (GenBank Accession Number X03205).

### Transient Transfection and Western analyses

For transfection, HepG2 cells were seeded at 1 ×10^6^ cells in a 60mm plate. Next day, cells were transfected with 6 μg of empty vector (pcDNA3.1) (Invitrogen, Waltham, MA, USACarlsbad, CACarlsbad, CA) or a plasmid (pcDNA3.1-HA-SHP) encoding HA tagged human SHP using PolyMAG (Chemicell, Berlin, Germany) [34]. For preparation of whole cell extracts the cells were washed twice with ice-cold PBS and harvested, then lysed with 50 mM Tris pH 7.4 RIPA buffer containing 150 mM NaCl, 1% NP-40, 0.5% Deoxycholate, 0.1% SDS, 0.5 mM PMSF, 1 g/ml Leupeptin, 1 g/ml Aprotinin, 50 mM β-Glycerophosphate, 25 mM NaF, 20 mM EGTA, 1 mM DTT, 1 mM Na3VO4 for 1 hour in ice. These lysates were centrifuged at 13,000 x g for 10 min at 4°C. The protein concentration of the supernatant was determined by using bradford assay (Bio-rad, Hercules, CA, USA). Equal amount of each protein lysate was separated by 7 ~ 9% SDS-PAGE and transferred to Polyvinylidene fluoride (PVDF) membrane by semi-dry by using Trans-Blot SD (Bio-Rad). The membrane was blocked with 5% skim milk in Tris-buffered saline containing tween-20 for 2 hr at room temperature. The membrane was incubated with primary antibodies for overnight at 4°C, followed by incubation with horseradish peroxidase (HRP) conjugated secondary antibodies. Proteins were visualized by an enhanced chemiluminescent (ECL) substrate kit (GE healthcare, Pittsburgh, PA, USA)

### Statistical analysis

The *p* value was analyzed by two-tailed Student’s *t*-test. All error bars represents the standard error of the mean (S.E.M.). Three qRT-PCR analyses were performed for an individual RNA sample. The experiments were repeated two to three times, thus six to nine values of qPCR were used for statistical analyses.

## Results

### CDCA represses endogenous HIF-1α target genes

In order to test effects of CDCA on the expression of HIF-1α target genes, HepG2 cells were serum starved with medium containing 0.5% FBS prior to CDCA treatments. After 6 hour treatments of CDCA, the cells were exposed to hypoxia (Fig 1A). We confirmed that CDCA increased SHP expression (Fig 1B and 1C). CDCA reduced hypoxic induction of carbonic anhydrase 9 (CA9) and phosphoglycerate kinase1 (PGK1) which are involved in pH balance and glycolysis respectively (Fig 1D). CDCA also reduced hypoxic induction of ERO1L which increases VEGF secretion. CA9, PGK1 and ERO1L are upregulated in many cancers. CDCA also reduced hypoxic induction of other HIF-1α target genes such as lysyl oxidase (LOX) and collagen prolyl-4 hydroxylase A1 (P4HA1) which are involved in hepatic fibrosis (Fig 1D).

### CDCA reduced the amount of HIF-1α protein

**Fig 2:**
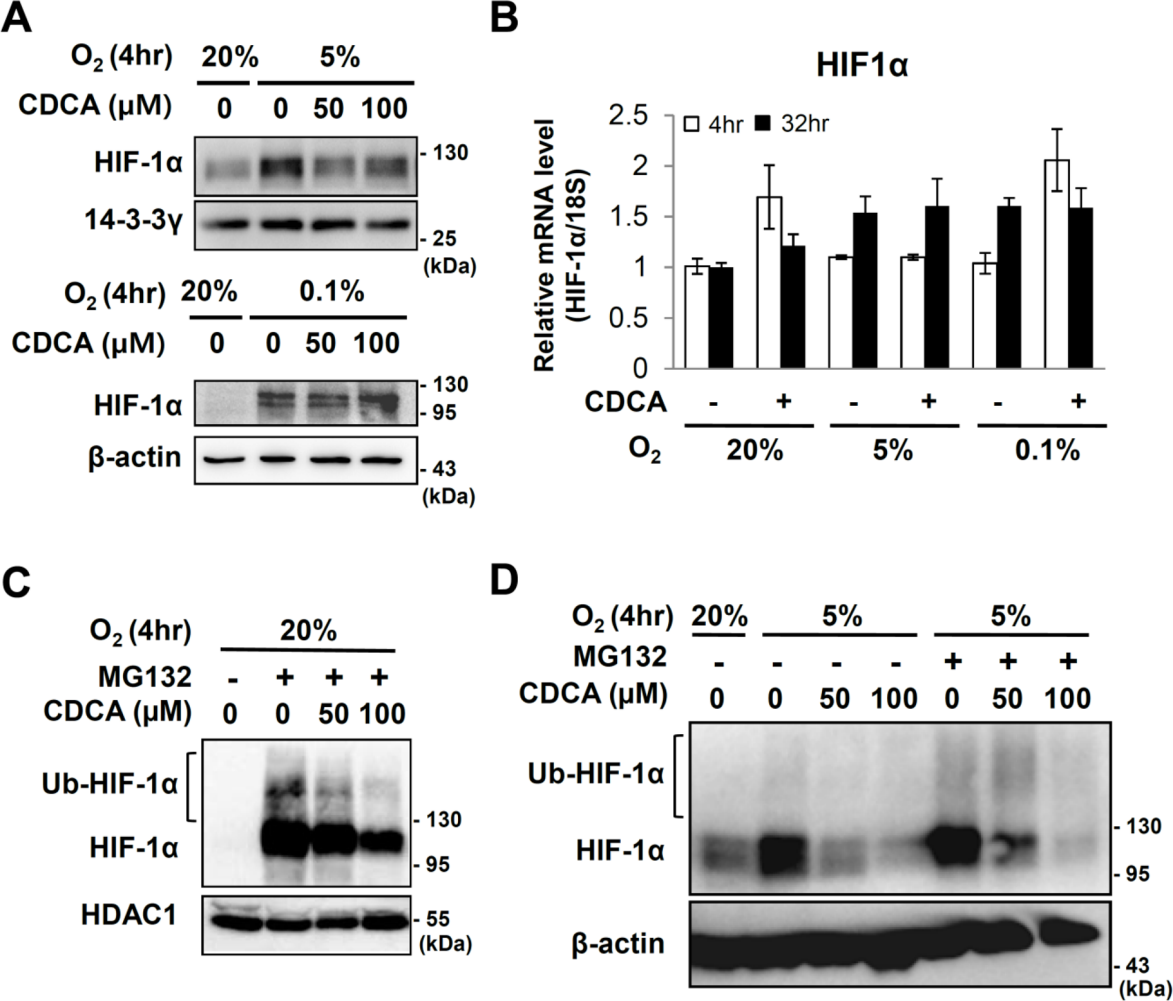
Effect of CDCA on HIF-1α expression. After starvation for 20hr, HepG2 cells were pretreated with CDCA (100 μM or indicated dose) for 6 hours then exposed to 20%, 5% or 0.1% O_2_ for the indicated hours. (A) Western analyses for HIF-1α, 14-3-3γ and β-actin proteins. 14-3-3γ and β-actin proteins were detected as loading controls. (B) Quantitative RT-PCR of HIF-1α mRNA. (C and D) Western analyses of HIF-1α protein. HepG2 cells which were serum starved with medium containing 0.5% FBS for 20 hours prior to stimulation with MG132 (10 μM) and/or CDCA. 6 hours after treatment, the cells were exposed to 20% or 5% O_2_ for 4 hours. Ubiquitinated and original HIF-1α proteins are indicated. HDAC1 protein or β-actin protein were examined in order to verify equal loading. (D) 20 μg of MG132 untreated total cell extracts and 5 μg of MG132 treated total cell extracts are loaded, respectively.

In order to determine whether or not CDCA changed the amount of HIF-1α protein, we exposed HepG2 cells to hypoxia (0.1% and 5% O_2_) in the presence of CDCA, and then detected the HIF-1α protein by western analyses. The results in Fig 2A showed that physiological hypoxia (5% O_2_) stabilized the HIF-1α protein, but which was reduced by CDCA treatment without significant changes in mRNA levels of HIF-1α. However, under severe hypoxia (0.1% O_2_) CDCA failed to reduce the levels of HIF-1α protein (Fig 2A and 2B). We tested whether or not CDCA enhances the degradation of HIF-1α protein. We treated HepG2 cells with MG132, an inhibitor of the 26S proteasome which protects ubiquitinated HIF-1α protein from degradation. Even under normoxia, MG132 stabilized the HIF-1α protein so that polyubiquitinated HIF-1α protein was observed as higher molecular weight bands. We found that even in the presence of MG132, CDCA reduced protein levels under conditions of both normoxia and physiological hypoxia (5% O_2_) (Fig 2C and 2D). These findings suggested that CDCA reduces the amount of HIF-1α protein not by enhancing degradation of HIF-1α protein.

### CDCA and SHP reduced the amount of HIF-1α protein in different mechanisms

**Fig 3:**
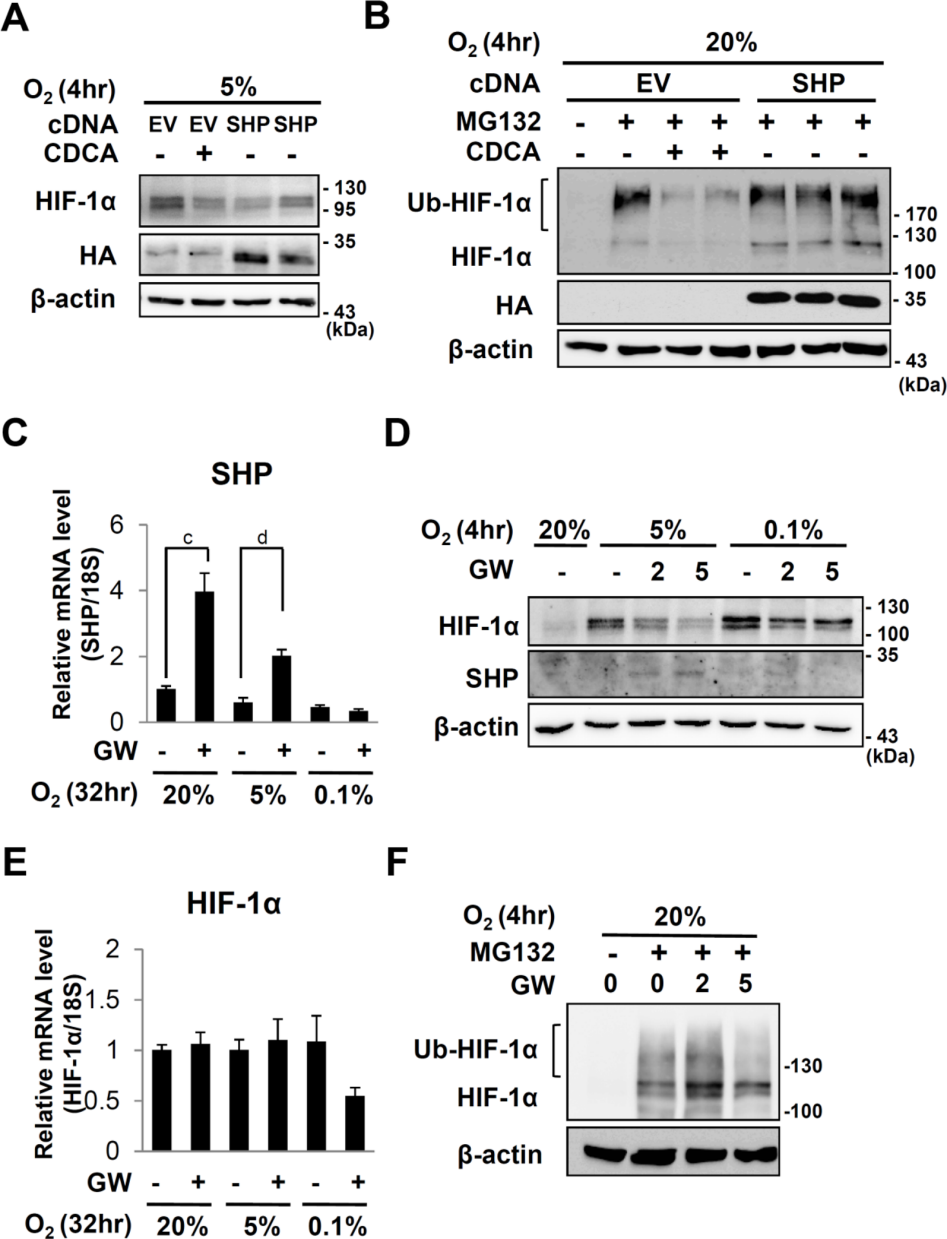
Effects of SHP or GW4064 on HIF-1α expression. (A and B) HepG2 cells were transfected with an empty vector or pcDNA3/HA-SHP. 18 hours after transfection, the cells were serum starved with medium containing 0.5% FBS for 20 hours. The cells were treated DMSO or 100 μM of CDCA for 6 hours in the absence or presence of MG132 and then exposed to 20%, 5% or 0.1% O_2_ for 4 hours. 30 μg of total cell extracts are loaded for western analyses. (C to E) After starvation for 20hr, HepG2 cells were pretreated with GW4064 (GW) (5 μM or indicated dose) for 6 hours then exposed to 20%, 5% or 0.1% O_2_ for the indicated hours. (C and E) qRT-PCR analyses of SHP or HIF-1α respectively. The expression level was normalized with the expression level of 18s rRNA. a, *p* ≤ 0.1; b, *p* ≤ 0.05; c, *p* ≤ 0.01; d, *p* ≤ 0.001. (D) Western analyses of HIF-1α, SHP and β-actin. (F) Western analyses of HIF-1α and β-actin in HepG2 cells which were treated with indicated doses of GW4064 and MG132 as described above. Ubiquitinated and original HIF-1α proteins are indicated. β-actin protein were examined in order to verify equal loading.

To test whether SHP, a target gene of CDCA also decreases HIF-1α protein, we transfected HepG2 cells with HA-tagged SHP, and then exposed to 5% O_2_. Overexpression of SHP reduced the amount of HIF-1α protein, even in the absence of CDCA (Fig 3A). Differently from CDCA, SHP overexpression failed to decrease HIF-1α protein in the presence of MG132 (Fig 3B). These results suggest that SHP decreased HIF-1α protein by enhancing its proteasomal degradation whereas CDCA reduced HIF-1α protein by recruiting additional mechanism which is not involved in proteasomal inhibition. The fact that CDCA can induce SHP implies that CDCA can reduce HIF-1α protein by at least two different mechanisms, SHP-dependent and independent mechanisms. SHP is also induced by GW4064 which has been identified as an exogenous agonist for FXR [32]. GW4064 treatment increased SHP mRNA under either normoxia or physiological hypoxia (5% O_2_) but not under severe hypoxia (0.1% O_2_) (Fig 3C). Like CDCA, GW4064 reduces HIF-1α protein under physiological hypoxia (5% oxygen) but not under severe hypoxia (0.1% oxygen) without changing mRNA levels of HIF-1α (Fig 3D and 3E). However differently from CDCA, GW4064 fails to decrease HIF-1α protein in the presence of MG132 (Fig 3F).

### Guggulsterone, a FXR antagonist also reduced HIF-1α protein

**Fig 4:**
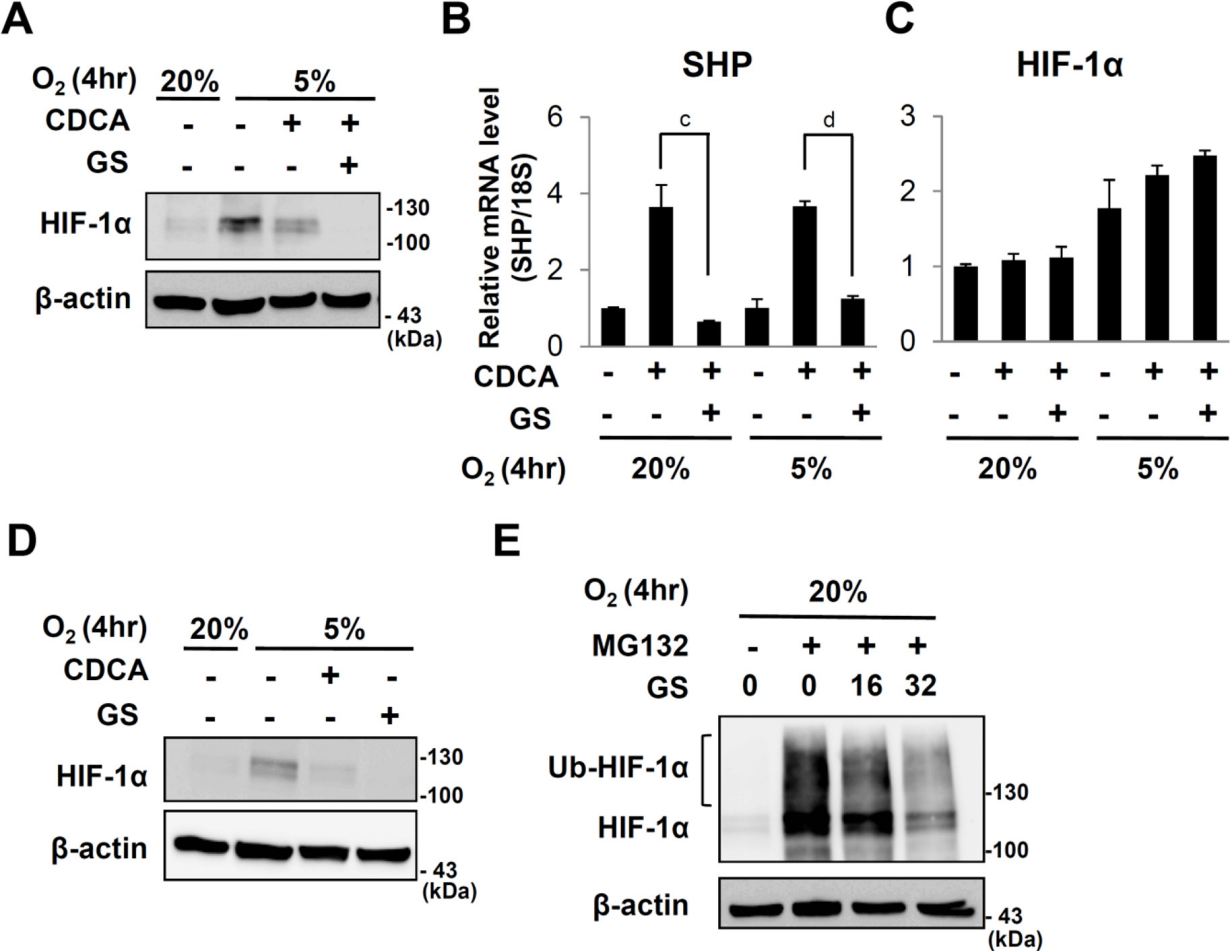
Effect of guggulsterone on HIF-1α expression. After starvation for 20hr, HepG2 cells were pretreated CDCA (100μM) and/or guggulsterone (GS) (32μM) for 6 hours then exposed to 20%, 5% or 0.1% O_2_ for 4 hours. (A) Western analyses of HIF-1α and β-actin. (B and C) qRT-PCR analyses of SHP and HIF-1α, respectively. c, *p* ≤ 0.01; d, *p* ≤ 0.001. (D) Western analyses of HIF-1α and β-actin in HepG2 cells which were pretreated with CDCA (100μM) and/or guggulsterone (32μM) for 6 hours and exposed to 20%, 5% or 0.1% O_2_ for 4 hours. (E) Western analyses of HIF-1α and β-actin in HepG2 cells which were pretreated with MG132 (10μM), CDCA (100μM) andor guggulsterone (32μM) for 6 hours and exposed to 20%, 5% or 0.1% O_2_ for 4 hours.

These results indicated that CDCA can reduce HIF-1α protein by recruiting different mechanism which is not related to SHP induction. Consistent with this idea, we found that guggulsterone, an antagonist of FXR did not reverse CDCA effects on HIF-1α, even though it prevents CDCA from inducing SHP expression. Instead guggulsterone and CDCA additively reduced HIF-1α protein levels under physiological hypoxia without significant changing mRNA levels of HIF-1α (Fig 4A to 4C). Furthermore, guggulsterone by itself reduced HIF-1α protein levels (Fig 4D). Like CDCA, guggulsterone also reduced HIF-1α protein in the presence of MG132 (Fig 4E).

### CDCA decreases de novo synthesis of HIF-1α protein

**Fig 5:**
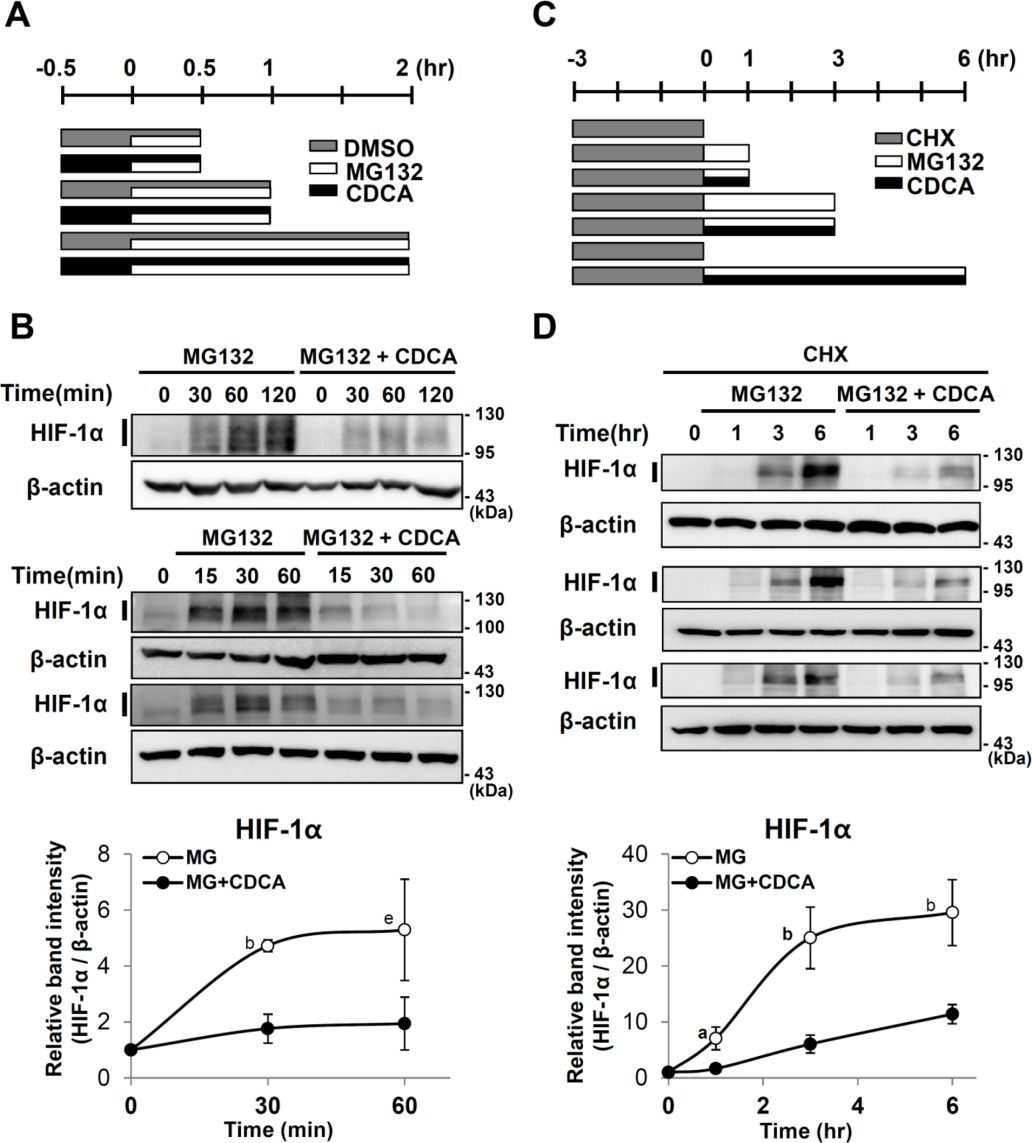
Effect of CDCA on de novo synthesis of HIF-1α protein. (A) Experimental scheme. After serum starvation for 20 hr, HepG2 cells were pretreated with DMSO or CDCA (100μM), 30 min prior to MG132 (10 μM) treatment. (B) Western analyses of HIF-1α and β-actin proteins (three western blots are shown). (C) Experimental scheme. HepG2 cells were pretreated with CHX (10 μg/ml, 3 hr), then the culture media were replaced with fresh media containing MG132 (10 μM) and/or CDCA (100 μM) as indicated. (D) Western analyses of HIF-1α and β-actin proteins (three western blots are shown). Quantification of western analyses. The intensities of HIF-1α and β-actin bands marked with bars were measured using Image J software. The y-axis indicates the relative band intensities of HIF-1α protein to 0 hr. The band intensities of HIF-1α protein were normalized by β-actin protein. The x-axis indicates the hours for MG132 treatments. *p* values between band intensities of CDCA-treated and untreated samples are shown. a, *p* ≤ 0.1; b, *p* ≤ 0.05; c, *p* ≤ 0.01; d, *p* ≤ 0.001; e, *p* = 0.251.

To test whether CDCA reduces synthesis of HIF-1α protein, we blocked HIF-1α degradation using MG132 in the presence or absence of CDCA under normoxia, then analyzed HIF-1α protein accumulation at different time points (Fig 5A). MG132 increased the accumulation of HIF-1α protein levels. However, in CDCA treated cells, the accumulation rate of HIF-1α protein was slower than in untreated cells (Fig 5B). To confirm that CDCA down-regulates *de novo* synthesis of HIF-1α protein, HepG2 cells were pretreated with a translation inhibitor, cyclohexamide (CHX) for 3 hours (Fig 5C). In the presence of CHX, HIF-1α protein was not detected (lane 1 in Fig 5D). After CHX containing culture media were replaced with fresh media, the newly translated HIF-1α protein was detected in the presence of MG132 (Fig 5D). CDCA treatments reduced the amount of the newly synthesized HIF-1α protein. These results suggest that CDCA decreases *de novo* synthesis of HIF-1α protein.

## Discussion

This study first tested potential HIF-1α inhibitors in different hypoxic conditions, physiological hypoxia (5% O_2_) and severe hypoxia (0.1% O_2_). Here we found that HIF-1α stabilized under physiological hypoxia is more susceptible to inhibitors. This finding reflects that if any, activators of PHD2 can hardly enhance its catalytic activities under severe hypoxia in which its substrate O_2_ is absent, and that severe hypoxia or anoxia changes synthesis mechanism of HIF-1α protein from Cap-dependent to Cap-independent translation. We found that CDCA reduced the amount of HIF-1α protein only under physiological hypoxia. By using a proteasome inhibitor MG132 and a translation inhibitor cyclohexamide, we found that CDCA reduced HIF-1α protein by decreasing its translation but not by enhancing its degradation. Although it remains to further investigate the molecular mechanism how CDCA decreases translation of HIF-1α, our findings indicated that FXR, a CDCA receptor and its target gene SHP are not involved in this effects of CDCA. Distinctly from CDCA, both GW4064 an exogenous FXR agonist and ectopic expression of SHP reduced HIF-1α protein by enhancing its degradation under physiological hypoxia. Furthermore CDCA and guggulsterone additively reduced HIF-1α protein, although guggulsterone prevents CDCA from inducing SHP. Interestingly, guggulsterone by itself reduced HIF-1α protein even in the presence of MG132. These findings suggested that CDCA and guggulsterone reduced the translation of HIF-1α in a mechanism which FXR and SHP are not involved. This study reveals novel therapeutic functions of these traditional safe drugs, CDCA and guggulsterone, as inhibitors of HIF-1α protein. Together with these findings, the facts that HIF-1α induces numerous hypoxic target genes which are involved in hepatic inflammation, cirrhosis and cancer imply that CDCA and guggulsterone can alleviate such hepatic diseases by inhibiting HIF-1α.

Many studies have revealed that HIF-1α plays important roles in tumor development, inflammatory metabolic diseases and fibrosis [21, 36, 37]. Therefore HIF inhibitors appear to have therapeutic effects on metabolic diseases and cancer. YC-1, temsirolimus also known as CCI-779 and Camptothecins have been reported as HIF-α inhibitors [38–41]. These inhibitors do not completely block hypoxic induction of HIF-1α target genes. Here we first revealed that CDCA and guggulsterone which has been therapeutically used to treat cholestatic liver diseases and colon cancer, directly reduced HIF-1α protein.

In liver parenchyma, metabolic enzymes and capacities are zonated because the composition of the blood changes during its passage from the periportal to perivenous areas. Of the many important components of blood, the oxygen gradient is a key regulator for the capacity of oxidative metabolism and induction of metabolic enzymes. The intracellular pO_2_ in the liver is about 45 to 50 mmHg (approximately 6% O_2_) in the periportal and 15 to 20 mmHg (approximately 2% O_2_) in the perivenous area. The capacity for bile formation is higher in the periportal area, while the capacity for glycolysis is greater in the more hypoxic perivenous area. Together with our findings, this relation between liver metabolism and pO_2_ infers that more bile acids produced in periportal area can reduce HIF-1α protein, leading to further decrement of HIF-1α target genes such as many glycolytic enzymes in periportal area [23, 42].

Pathologically, ischemia-reperfusion injury occurs in the context of reperfusion of the transplanted liver and with low arterial pressure. Many studies indicated that HIF-1α has protective effects on ischemia-reperfusion injury [43]. Hypoxia is also developed in liver exposed to alcohol because both chronic and acute alcohol increases oxygen consumption in liver by inducing many alcohol detoxifying enzymes [44]. However, protective role of HIF-1α is controversial. Chronic alcohol administration increased hepatic steatosis in an HIF-1α dependent manner [27]. Recent studies described the effect of HIF-1α deletion in bile duct ligated (BDL) mice, an animal model of cholestatic liver injury. Both wild type and HIF-1α-deleted mice show increase in serum bile acids, but HIF-1α deleted mice were protected from increases in liver fibrosis [43, 45, 46]. In hepatocellular carcinoma, several genes which were differentially regulated by chronic hypoxia showed relationship with poor prognosis [47]. Our finding that CDCA, guggulsterone reduced amount of HIF-1α protein suggests that these can alleviate the hepatic fibrosis and cancer which are enhanced by HIF-1α.

We found that CDCA reduced HIF-1α protein by inhibiting the translation of HIF-1α. Under physiological hypoxia or normoxia, HIF-1α is translated by Cap-dependent mechanism. It was reported that a variety of growth factors and cytokines increases Cap-dependent translation of HIF-1α through PI3 kinase/mTOR pathway. This pathway leads to phosphorylation of 4E-binding protein1 (4E-BP1) and p70SK kinase (p70S6K). 4E-BP1 blocks the formation of translation initiation complex but phosphorylated 4E-BP1 fails to do so. Phosphorylated p70S6K becomes activated to promote Cap-dependent translation [48]. However severe hypoxia (< 1% oxygen) inhibits Cap-dependent translation leading to shutdown of general protein translation by two mechanisms; i.e., activation of unfolded protein response (UPR) and inhibition of mTOR pathway. Severe hypoxia (< 1%) triggers UPR by activating the endoplasmic reticulum kinase, PKR-like ER kinase (PERK) which phosphorylates and inhibits eIF2α, a key factor for translation initiation. Prolonged hypoxia or nutrient depletion activates AMP kinase which inhibits mTOR pathway. Despite shutdown of general protein translation, severe hypoxia maintains translation of HIF-1α through Cap-independent mechanism. During hypoxia, the RNA-binding proteins, poly pyrimidine tract-binding protein (PTB) and HuR bind the HIF-1α mRNA thereby enhancing HIF-1α translation [49–53]. Hypoxia activates 4E-BP1 and eIF4G that facilitate cap-independent translation over cap-dependent translation of HIF-1α [48].

A few HIF-1α inhibitors have been identified to reduce the translation of HIF-1α. A nontoxic flavonoid Silibinin which is an active component of milk thistle suppressed HIF-1α translation by inhibiting mTOR signaling. Mitochondrial dysfunction by using mitochondrial inhibitors and an uncoupler suppressed HIF-1α protein synthesis through activation of AMPK [54].

This study showed that CDCA and guggulsterone reduced the synthesis of HIF-1α in a mechanism which is not associated with FXR and SHP. It remains to investigate whether CDCA and guggulsterone intervene in Cap-dependent HIF-1α translational regulatory pathway.
